# Isolated medial patellofemoral ligament reconstruction for recurrent patellofemoral instability: analysis of outcomes and risk factors

**DOI:** 10.1186/s13018-021-02383-9

**Published:** 2021-04-06

**Authors:** Filippo Migliorini, Francesco Oliva, Gayle D. Maffulli, Jörg Eschweiler, Matthias Knobe, Markus Tingart, Nicola Maffulli

**Affiliations:** 1 Department of Orthopaedics, RWTH Aachen University Clinic, Pauwelsstraße 30, 52074 Aachen, Germany; 2 Department of Medicine, Surgery and Dentistry, University of Salerno, Via S. Allende, 84081 Baronissi, SA Italy; 3 Wholelife Clinics, London, UK; 4 Department of Orthopedics and Trauma Surgery, Lucerne Cantonal Hospital, Lucerne, Switzerland; 5 Barts and the London School of Medicine and Dentistry, Centre for Sports and Exercise Medicine, Queen Mary University of London, Mile End Hospital, 275 Bancroft Road, London, E1 4DG UK; 6 School of Pharmacy and Bioengineering, Keele University Faculty of Medicine, Thornburrow Drive, Stoke on Trent, UK

**Keywords:** Patellofemoral instability, MPFL reconstruction, Risk factors

## Abstract

**Background:**

The medial patellofemoral ligament (MPFL) is always damaged after patellar dislocation. In selected patients, MPFL reconstruction is necessary to restore a correct patellar tracking. Despite the large number of different techniques reported to reconstruct the MPFL, there is no consensus concerning the optimal procedure, and debates is still ongoing. The present study analysed the results after isolated MPFL reconstruction in patients with patellofemoral instability. Furthermore, a subgroup analysis of patients presenting pathoanatomical risk factors was made.

**Methods:**

In November 2020, the main electronic databases were accessed. All articles reporting the results of primary isolated MPFL reconstruction for recurrent patellofemoral instability were considered for inclusion. Only articles reporting a minimum 12-month follow-up were eligible.

**Results:**

Data from a total of 1777 knees were collected. The mean age of the patients involved was 22.8 ± 3.4 years. The mean follow-up was 40.7 ± 25.8 months. Overall, the range of motion (+ 27.74; *P* < 0.0001) and all the other scores of interests improved at last follow-up: Kujala (+ 12.76; *P* = 0.0003), Lysholm (+ 15.69; *P* < 0.0001), Tegner score (+ 2.86; *P* = 0.006). Seventy-three of 1780 patients (4.1%) showed a positive apprehension test. Thirty of 1765 patients (1.7%) experienced re-dislocations, while 56 of 1778 patients (3.2%) showed persisting joint instability. Twenty-five of 1786 patients (1.4%) underwent revision surgeries.

**Conclusion:**

Isolated MPFL reconstruction for recurrent patellofemoral instability provides reliable surgical outcomes. Patients with pathoanatomical predisposing factors reported worse surgical outcomes.

## Introduction

Recurrent patellofemoral instability is common, accounting for approximately one-third of all knee injuries in sports medicine [1, 2]. Patients suffering from recurrent patellofemoral instability frequently present underlying pathoanatomical abnormalities which predispose them to patellar dislocation [3, 4]. These alterations incorporate bony conformation abnormalities including trochlear dysplasia [5], lower limb mal-alignment syndromes such as tibial extra-rotation [6] and soft tissue abnormalities such as patella alta [7]. Most patients present with a combination of two or more concomitant pathoanatomical risk factors [8, 9]. Given its multifactorial aetiology, the management of recurrent patellofemoral instability can be challenging [10–12]. In non-surgical treatment, most patients experience recurrent patella dislocations, pain and instability in the affected knee [13, 14], leading to a lower level of activity and reduced quality of daily living [15]. Hence, surgical reconstruction of the medial patellofemoral ligament (MPFL) represents a feasible option in those patients [9, 16, 17]. MPFL reconstruction shows an appreciable improvement in quality of life and recreational participation [18, 19]. As a result of the highly promising outcomes recorded, an isolated MPFL reconstruction can be performed even in patients presenting with low- to moderate-grade pathoanatomical alteration, avoiding bony interventions [20–22]. The number of different procedures described to reconstruct the MPFL in these patients is increasing exponentially, and assessment of these options has become a point of considerable research interest [23, 24]. However, there has been no consensus concerning results, and debates are still ongoing [25–27].

Thus, we conducted a systematic review of the literature to analyse results after isolated MPFL reconstruction in patients with recurrent patellofemoral instability. Furthermore, we performed subgroup analyses of patients presenting pathoanatomical risk factors. The focus of the present systematic review was on clinical scores and examinations, rate of revision surgeries, re-dislocations and persistent joint instability. We hypothesised that this procedure provides reliable surgical outcomes and that patients with predisposing risk factors are more prone to complications.

## Materials and methods

### Search strategy

A comprehensive review of the literature was performed according to the Preferred Reporting Items for Systematic Reviews and Meta-Analyses guidelines (PRISMA) [28]. To guide the search, a preliminary protocol was defined:

- Population: recurrent patellofemoral instability;
- Intervention: primary isolated MPFL reconstruction;
- Outcomes: clinical scores, physical examination, complications;
- Timing: > 24 months follow-up.

### Literature search

Two independent reviewers (**;**) performed the search separately. The following electronic databases were accessed: PubMed, Medline, Embase and Google Scholar. In November 2020, the following terms were used in combination: *knee*, *patellofemoral*, *dislocation*, *recurrent*, *instability*, *medial patellofemoral ligament*, *MPFL*, *tear*, *rupture*, *surgery*, *reconstruction*, *TT*-*TG*, *trochlear*, *dysplasia*, *patella alta*, *apprehension test*, *Kujala*, *Lysholm*, *Tegner*, *re*-*dislocation*, *failure*, *reoperation*, *revision*, *subluxation.* If title and abstract matched the topic, the full-text was accessed. Furthermore, a cross reference of bibliographies was performed to improve the studies for inclusion. Disagreements between the authors were debated and solved by a third author (**).

### Eligibility criteria

All the articles treating primary isolated MPFL reconstruction for recurrent patellofemoral instability were considered for inclusion. According to the authors’ capabilities, articles published in English, French, German, Italian or Spanish were considered. Articles with level of evidence I to IV, according to the Oxford Centre of Evidence Based Medicine [28], were considered. Technical articles, comments, letters, editorials, protocols, guidelines and review articles were excluded. Biomechanical, animal and cadaveric studies were also excluded. Studies on MPFL reconstruction after total knee arthroplasty were excluded, as were articles reporting surgical outcomes regarding combined interventions were also rejected. Articles reporting MPFL rupture with direct suture, ligament plastic or medial retinaculum reefing were excluded. Articles treating MPFL reconstruction in a revision setting were also excluded, along with those treating acute injuries. Only articles reporting a minimum of 12-month follow-up were included. Only articles that reported quantitative data concerning the outcomes of interest were included. Missing data pertinent to these parameters warranted exclusion from this systematic review. The same investigators screened the articles for inclusion. A cross-reference research of the selected articles was performed to identify any article omitted from the initial search.

### Outcomes of interest

Two investigators (**;**) extracted the following data independently: patient demographics (author and year, number of procedures, mean age at time of surgery); mean follow-up duration; type of study. Patients clinical status has been evaluated through the analysis of three scores: the Kujala Anterior Knee Pain Scale [29], the Lysholm Knee Scoring Scale [30] and the Tegner Scale [31]. Range of motion (ROM) was also analysed. Postoperative complications were recorded for each publication: apprehension test, revision surgeries, re-dislocations and persistent joint instability. The latter was defined as recurrence and/or subjective sensation of subluxation or instability [32]. Furthermore, the presence of abnormal tibial tuberosity-trochlea groove distance (TT-TG) of patellar height and trochlear dysplasia was recorded.

### Methodological quality assessment

For the methodological quality assessment, we used the PEDro score. Two authors independently (**;**) performed the score calculation. The PEDro score analyses each included article under several items: allocation, presence of randomisation or blinding methods, clear inclusion and exclusion criteria, duration of follow-up and type of analysis. The final score ranks from 0 (poor quality) to 10 (excellent quality). Values > 6 points are considered as acceptable.

### Statistical analysis

The statistical analysis was performed through the software IBM SPSS. For continuous variables, the weighted mean differences and the standard deviation (SD) between groups were adopted. For binary variables, the odd ratio (OR) effect measure was used. The confidence interval was set at 95% in all the comparisons. Values of *P* < 0.05 were considered statistically significant.

## Results

### Literature search

**Fig. 1:**
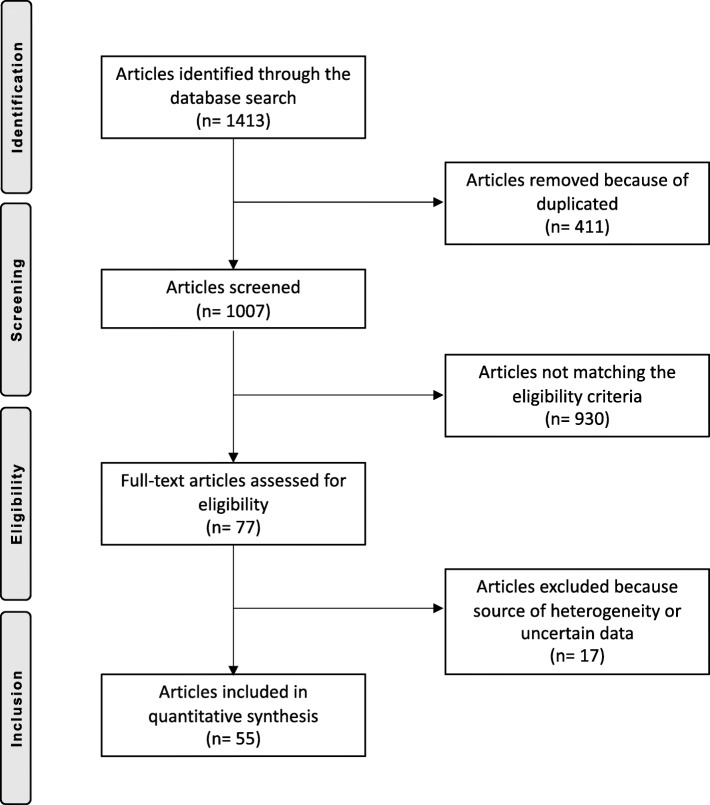
PRISMA flow-chart of the literature search

The literature search and cross-referencing resulted in a total of 1413 references, of which 411 were rejected because of duplications. Of these, another 930 were rejected or deviations from the eligibility criteria, leaving 77 publications for review. After reading the remaining full-text articles, another 17 articles were excluded, given insufficient details and/or uncertain diagnoses or outcome measures. Finally, 55 articles were included in the present analysis (Fig. 1).

### Methodological quality assessment

The PEDro score evidenced several limitations. First, only 7% (4/55) of article were randomised studies. None of the included studies used any blinding. Strength points were the number of prospective studies and the length of follow-up provided by most studies. In total, the PEDro score resulted in 6.38 ± 1.0 points, attesting to the quality of this systematic review. The results of the PEDro score assigned to each study are shown in Table 1.

**Table 1:** Demographics of studies included and related PEDro score. (N/R: not reported; Semit: semitendinosus; Ham: hamstring; Synth: synthetic; Add: adductor; Quad: quadriceps)

Author, year	Study design	Mean follow-up	PEDro score	Knees (*n*)	Mean age	Trochlear dysplasia	Patella Alta	Elevated TT-TG	Patellar bundle	Patellar fixation	Femoral fixation	Graft source
Amin et al. 2015 [33]	Retrospective	24	6	8	22.0	N/R	N/R	N/R	Single	Bone tunnel	Interference screw	Semit
Astur et al. 2015 [34]	Randomised	60	8	30	31.1	No	No	No	Single	Endobutton	Interference screw	Gracilis
				28	28.3	No	No	No	Double	Anchor	Interference screw	Gracilis
Ballal et al. 2018 [35]	Prospective	12	7	20	24.4	Yes	No	No	N/r	Suture anchor	Interference screw	Semit
Biondi Pinheiro et al. 2018 [25]	Retrospective	31	7	16	27.1	No	No	No	Single	Anchor	Interference screw	Semit
		35		21	26.4	No	Yes	No	Single	Anchor	Interference screw	Semit
Bitar et al. 2011 [36]	Randomised	24	8	21	N/R	N/R	N/R	N/R	Single	Patellar tendon pedicle	Interference screw	Patellar
Bitar et al. 2015 [37]	Retrospective	19	7	56	23.0	N/R	N/R	N/R	Double	Anchor	Interference screw	Gracilis
Calapodopulos et al. 2016 [38]	Prospective	30	5	22	23.1	Yes	No	No	Single	Quad tendon pedicle	Anchor	Quad
Christiansen et al. 2008 [39]	Prospective	22	6	32	22.0	N/R	N/R	N/R	Double	Bone tunnel	Interference screw	Gracilis
Csintalan et al. 2014 [40]	Retrospective	51	5	56	24.3	Yes	Yes	N/R	Double	Bone tunnel	Interference screw	Semit
Deie et al. 2011 [41]	Retrospective	39	5	31	22.2	Yes	No	No	Double	Soft tissue	Bone plug	Semit
Feller et al. 2014 [20]	Retrospective	42	5	26	24.4	No	No	No	Double	Bone tunnel	Interference screw	Hamstring
Fink et al. 2014 [42]	Prospective	12	7	17	21.5	Yes	N/R	No	Double	Quad tendon pedicle	Interference screw	Quad
Gomes et al. 1992 [43]	Retrospective	39	5	30	28.0	N/R	N/R	N/R	Single	Bone tunnel	Interference screw	Synth
Gomes et al. 2004 [44]	Prospective	60	6	16	26.7	Yes	Yes	No	Single	Bone tunnel	Soft tissue	Semit
Gomes et al. 2008 [45]	Prospective	53	7	12	19.3	N/R	Yes	N/R	Single	Bone tunnel	Soft tissue	Semit
				12	19.0	N/R	Yes	N/R	Single	Bone tunnel	Pedicled adductor magnus	Add Magnus
Goncaives et al. 2011 [46]	Prospective	26	6	22	28.6	No	No	No	Double	Bone tunnel	Interference screw	Semit
Han et al. 2011 [47]	Retrospective	68	6	59	24.3	No	Yes	No	Single	Bone tunnel	Interference screw	Semit
Hiemstra et al. 2017 [48]	Retrospective	24	5	155	25.4	Yes	Yes	Yes	Single	Suture anchor	Interference screw	Hamstring
Hinterwimmer et al. 2013 [49]	Retrospective	16	6	19	23.0	Yes	N/R	No	Double	Bone tunnel	Interference screw	Gracilis
Kang et al. 2013 [50]	Randomised	24	8	82	28.8	No	No	No	Double	Soft tissue	Interference screw	Semit
Kim et al. 2015 [51]	Retrospective	19	6	9	24.6	Yes	No	No	Mixed	Soft tissue	Suture anchor	Gracilis
Kita et al. 2015 [52]	Prospective	39	7	44	25.4	Yes	Yes	Yes	Double	Bone tunnel	Interference screw	Semit
Krishna Kumar et al. 2014 [53]	Prospective	25	7	30	18.0	No	N/R	No	Double	Endobutton	Interference screw	Gracilis
Li et al. 2014 [54]	Prospective	79	7	65	29.4	Yes	N/R	No	Double	Soft tissue	Interference screw	Tibialis Ant
Lind et al. 2016 [55]	Prospective	39	8	24	12.5	Yes	N/R	Yes	Double	Bone tunnel	Soft tissue	Gracilis
		41		179	23.0	Yes	N/R	No	Double	Bone tunnel	Interference screw	Gracilis
Lin et al. 2015 [56]	Retrospective	35	5	18	N/R	Yes	No	No	Double	Suture anchor	Interference screw	Semit
Lippacher et al. 2014 [57]	Retrospective	25	7	68	18.3	Yes	Yes	No	Double	Bone tunnel	Interference screw	Gracilis
Ma et al. 2013 [58]	Randomised	40	8	32	28.4	No	N/R	Yes	Double	Anchor	Interference screw	Semit
Matsushita et al. 2014 [26]	Retrospective	44	6	21	22.1	N/R	No	Yes	Double	Anchor	Interference screw	Semit
		38		18	23.5	N/R	No	No	Double	Anchor	Interference screw	Semit
Nomura et al. 2000 [59]	Prospective	71	7	27	21.0	No	Yes	No	Single	Bone tunnel	Interference screw	Synth
Nomura et al. 2007 [60]	Retrospective	143	5	24	22.5	Yes	Yes	No	Single	Bone tunnel	Staple	Synth
Panni et al. 2011 [61]	Retrospective	33	5	48	25.0	No	No	No	Double	Bone tunnel	Interference screw or anchor	Semit
Raghuveer et al. 2012 [62]	Prospective	42	7	15	29.2	No	No	No	Single	Bone tunnel	Interference screw or anchor	Semit
Ronga et al. 2009 [63]	Prospective	37	5	37	28.0	No	No	No	Double	Bone tunnel	Interference screw or anchor	Hamstring
Sadigursky et al. 2016 [64]	Prospective	12	7	31	29.4	Yes	Yes	No	Double	Anchor	Interference screw	Semit
Sillanpaa et al. 2008 [65]	Retrospective	121	6	18	20.2	Yes	Yes	Yes	Single	Soft tissue	Adductor pedicle	Add Magnus
Slenker et al. 2013 [66]	Retrospective	21	6	35	20.6	N/R	N/R	N/R	Single	Bone tunnel	Interference screw	Hamstring
Smith et al. 2014 [67]	Retrospective	12	6	21	23.0	No	N/R	N/R	Double	Bone tunnel	Interference screw	Hamstring
Suganuma et al. 2016 [68]	Retrospective	52	6	18	20.7	No	No	No	Doubled	Spike staple	Spike staple	Synth
		48		28	20.3	No	No	No	Doubled	Spike staple	Spike staple	Synth
Thaunat et al. 2007 [69]	Retrospective	28	5	23	22.0	Yes	Yes	No	Doubled	Bone tunnel	Suture anchor	Gracilis
Vavalle et al. 2016 [70]	Retrospective	38	5	16	22.0	Yes	No	No	Single	Quad tendon pedicle	Suture anchor	Quad
Wagner et al. 2013 [22]	Prospective	12	6	50	19.0	Yes	Yes	Yes	N/r	Suture anchor	Interference screw	Gracilis
Wang et al. 2010 [71]	Retrospective	42	7	28	29.0	Yes	No	No	Single	Suture	Interference screw	Semit
Wang et al. 2016 [72]	Retrospective	38	6	26	26.3	Yes	No	No	Single	Suture	Interference screw	Semit
Wantabe et al. 2017 [73]	Retrospective	52	7	29	19.0	N/R	N/R	N/R	Double	Suture anchor	Interference screw	Gracilis
Witonski et al. 2013 [74]	Prospective	43	7	10	27.2	No	No	No	Double	Suture anchor	Endobotton	Hamstring
Zhang et al. 2019 [75]	Prospective	96	7	60	21.0	No	No	No	N/r	Patellar tendon pedicle	Suture anchor	Patellar

### Demographic data

Data from a total of 1777 knees were collected. The mean age of the patients was 22.8 ± 3.4 years. The mean follow-up was 40.7 ± 25.8 months. Twenty-three of 55 articles (42%) reported data of patients with imaging evidence of trochlear dysplasia, 7 of 55 (13%) with elevated TT-TG and 15 of 55 (27%) with patella alta. Further, 33.8% (606 of 1795) of procedures were performed using a single bundle patellar graft insertion, while 66.2% though a double bundle. Patellar fixation was achieved through a bone tunnel in 44.5% (837 of 1884) of procedures, suture anchors in 30.8% (581 of 1884), soft tissue procedures 10.9% (205 of 1884), suture 23.6% (69 of 1884), Endobutton 3.2% (60 of 1884), quadriceps tendon pedicle 2.9% (55 of 1884), staple 2.4% (46 of 1884) and patellar tendon pedicle 1.6% (31 of 1884). Femoral fixation was achieved though interference screw 83.6% (1492 of 1874), anchors 4.5% (80 of 1874), staple 9.2% (70 of 1874), soft tissue procedures 2.9% (52 of 1874), bone plug 1.7% (31 of 1874), adductor pedicle 1.7% (30 of 1874) and Endobutton 1.6% (29 of 1874). Semitendinosus was used in 37.0% (699 of 1884) of procedures, gacilis 30.5% (574 of 1884), synthetic 6.7% (127 of 1884), quadriceps 2.9% (55 of 1884), patellar 31% (1.6 of 1884) and adductor magnus 30% (1.6 of 1884). The demographic data of studies included are shown in Table 1.

### Clinical findings

Overall, the ROM (+ 27.74; *P* < 0.0001) and all the other scores of interests improved at the last follow-up: Kujala (+ 12.76; *P* = 0.0003), Lysholm (+ 15.69; *P* < 0.0001), Tegner score (+ 2.86; *P* = 0.006). These results are shown in detail in Table 2.

**Table 2:** Analyses of the endpoint: clinical scores

Endpoint	Pre-operative	Post-operative	Improvement	P
Kujala	75.54 ± 9.7 (67.3 to 81.8)	88.30 ± 5.9 (97.7 to 71)	12.76	0.0003
Lysholm	74.41 ± 9.6 (59.1 to 80.4)	90.10 ± 4.0 (96.4 to 79.7)	15.69	> 0.0001
Tegner	2.43 ± 2.2 (1.1 to 3.9)	5.29 ± 1.0 (7.82 to 4)	2.86	0.006
Rom	105.31 ± 25.3 (94.6 to 118.1)	133.05 ± 9.0 (141.3 to 125.9)	27.74	> 0.0001

### Complications

Seventy-three of 1780 patients (4.1%) showed a positive apprehension test. Thirty of 1765 patients (1.7%) experienced re-dislocations, while 56 of 1778 patients (3.2%) showed persisting joint instability. Twenty-five of 1786 patients (1.4%) underwent further revision surgeries.

### Subgroup analyses

The presence of pathoanatomical risk factors do not influence the Kujala, Lysholm and Tegner scores, as did the rate of positiveness to the apprehension test (Table 3).

**Table 3:** Subgroup analyses of the endpoint: clinical scores

Endpoint	Normal range	Abnormal range	*P*
Physiological TT-TG			
Kujala	87.95 ± 5.9	85.32 8.4	0.2
Lysholm	88.59 ± 3.9	86.92 4.1	0.3
Tegner	5.58 ± 1.1	4.50 0.7	0.1
Physiological patellar height			
Kujala	88.36 ± 5.8	88.12 5.7	0.9
Lysholm	88.87 ± 3.6	85.67 8.4	0.4
Tegner	5.82 ± 1.3	4.88 1.1	0.3
Physiological trochlea morphology			
Kujala	87.51 ± 5.4	87.31 8.5	0.5
Lysholm	91.64 ± 4.0	88.38 3.9	0.2
Tegner	4.83 ± 1.1	5.51 1.0	0.2

Studies treating patients within the normal range of TT-TG distance reported a lower rate of revision surgeries (OR: 0.09; 95% CI: 0.0302 to 0.2943; *P* < 0.0001), re-dislocations (OR: 0.2; 95% CI: 0.0754 to 0.3669; *P* < 0.0001) and persistent join instability (OR: 0.3; 95% CI: 0.1660 to 0.5886; *P* = 0.0003) compared to those treating patients with an increased TT-TG. Studies treating patients with patella height within the normal range reported a lower rate of revision surgeries (OR: 0.8; 95% CI: 0.1495 to 1.6667; *P* = 0.04), re-dislocations (OR: 0.2; 95% CI: 0.0514 to 0.6044; *P* = 0.006) and persistent joint instability (OR: 0.2; 95% CI: 0.0929 to 0.4825; *P* = 0.0002) compared to those treating patients with signs of patella alta. Studies treating patients with trochlear morphology within the normal anatomic range reported a lower rate of revision surgeries (OR: 0.2; 95% CI: 0.0536 to 0.6541; *P* = 0.009), re-dislocations (OR: 0.2; 95% CI: 0.0503 to 0.4216; *P* = 0.0004) and persistent joint instability (OR: 0.2; 95% CI: 0.0832 to 0.3860; *P* < 0.0001) compared to those treating patients with of trochlear dysplasia. These results are shown in detail in Table 4.

**Table 4:** Subgroup analyses of the endpoint: complications

Endpoint	Odd Ratio	95% CI	*P*
Physiological TT-TG			
Apprehension	0.9	0.6874 to 1.0002	0.8
Joint instability	0.3	0.1660 to 0.5886	0.0003
Re-dislocations	0.2	0.0754 to 0.3669	< 0.0001
Revision surgeries	0.09	0.0302 to 0.2943	< 0.0001
Physiological patellar height			
Apprehension	0.8	0.7465 to 1.0432	0.9
Joint instability	0.2	0.0929 to 0.4825	0.0002
Re-dislocations	0.2	0.0514 to 0.6044	0.006
Revision surgeries	0.8	0.1495 to 1.6667	0.04
Physiological trochlea morphology			
Apprehension	0.9	0.5230 to 1.1039	0.8
Joint instability	0.2	0.0832 to 0.3860	< 0.0001
Re-dislocations	0.2	0.0503 to 0.4216	0.0004
Revision surgeries	0.2	0.0536 to 0.6541	0.009

## Discussion

The present study assessed the outcome of isolated MPFL reconstruction in selected patients with patellar instability. Isolated MPFL reconstruction for recurrent patellofemoral instability provided very good outcomes, as witnessed by the Kujala, Lysholm and Tegner scores. Patients with patella alta, trochlear dysplasia and those with elevated of TT-TG distance showed an increased rate of revision surgeries, re-dislocations and persistent joint instability compared to those without the presence of pathoanatomical risk factors. Patients with elevated TT-TG distance are more prone to revision surgery.

The MPFL is the most important static restraint to the lateral displacement of the patella during the first 30° of flexion [76]. After the first patellar dislocation, the MPFL is always damaged [77, 78] and a ligament reconstruction is often required [12, 79]. Patients without imaging evidence of pathoanatomical risk factors are suitable for isolated MPFL reconstruction [80–83]. Patellar instability is a multifactorial condition [20, 84, 85]. Twenty-six percent of the patients had two, about 17% three and 15% four concomitant risk factors [86]. Other imaging studies detected similar observations [8, 9]. Thus, proper treatment consists of adequate analysis of the associated pathoanatomical risk factors prior to MPFL reconstruction [87]. The question worth discussing remains whether isolated MPFL reconstruction alone or combined with other procedures are needed to restore optimal biomechanics and patellar tracking [20]. Regarding preoperative planning of such procedures, no clear or detailed recommendations have been established. Each patient must be evaluated individually and the decision to combine reconstruction of the MPFL with another procedure still depends on the clinical judgement of the treating orthopaedic surgeon.

Patients presenting with high-grade of pathoanatomical risk factors are likely suitable for combined interventions. Combining different interventions aiming to stabilise the extensor mechanism most probably prevents further complications such as soft tissue damage or long-term degenerative joint disease such as osteoarthritis, while also improving quality of life and activity level [84, 88]. In the present study, the outcomes of studies reporting the results of MPFL reconstruction in patients presenting pathoanatomical risk factors were also compared.

Analysing data from studies reporting MPFL reconstruction in patients with low-grade trochlear dysplasia showed no evidence of a statistically significant association with clinical scores or clinical examination, but nevertheless represent an increased risk to incur revision surgeries, re-dislocations and persistent joint instability. The most common intervention to restore roughly correct patellar tracking are an opening wedge osteotomy according to Albee [89] and the sulcus deepening trochleoplasty [90]. These procedures are associated with controversial outcomes, and performing opening wedge osteotomies must be considered with caution [91, 92]. Evidence concerning sulcus-deepening trochleoplasty is limited [93–95]. Wagner et al. [22] found worse results with isolated MPFL reconstruction in patients with severe trochlear dysplasia. Steiner et al. [96] performed an isolated MPFL reconstruction in patients with low- to severe-grade of trochlear dysplasia, and reported no dislocation at a minimum of 24-month follow-up. Recently, Kohn et al. [97] analysed the outcomes of isolated MPFL reconstruction in patients with high-grade trochlear dysplasia. The degree of trochlear dysplasia which might require an isolated MPFL reconstruction only remains unclear [97].

Studies including patients with a low grade of increased TT-TG and patella alta showed no difference in the clinical scores and clinical examination, but a moderate risk of subsequent re-dislocations and persistent joint instability. Interestingly, the analysis of the rate of revision surgeries was high in the elevated TT-TG but low in the patella alta group. These data require further investigations.

To evaluate patella alta, the most common indexes are the Caton-Deschamps [98] and/or the Install-Salvati ratio [99], while a suitable method to rate the trochlear dysplasia is the classification of Dejour et al. [100]. The TT-TG distance is used to investigate the tibial extra-rotation over the femoral axis [21]. These pathoanatomical risk factors with related rating index were not quantitatively evaluated by most of the included studies. Most of studies referred to low- to severe-grade of alteration, without proper data quantification. Therefore, these pathoanatomical risk factors could not be analysed in a quantitative fashion. This represents an important limitation of this study. The poor quality of most of the articles included constitutes another notable limitation. Lack of high-quality comparative trials is prevalent, therefore significantly reducing the overall evidence and opportunity to conduct a high-quality review. Surgical protocols for MPFL reconstruction and post-operative rehabilitation were not considered in the present investigation. The latter were heterogeneous throughout all the included studies. This therefore represents a potential source of bias. Indeed, approach, procedures and grafts were heterogeneous, as were the rehabilitation protocols. Given these limitations, data from the present study must be interpreted with caution. Points of strength of this systematic review are the comprehensive nature of the literature search along with the strict eligibility criteria. The methodological assessment resulted in a good quality assessment, and the baseline of samples was comparable, representing another strength point. Most studies reporting data from patients with additional previous surgeries did not clarify the nature of the interventions. Therefore, further considerations were not possible. Future studies should be aimed to clarify the role of other important risk factors, such as genu valgum, patellar dysplasia and femoral anteversion.

## Conclusion

Isolated MPFL reconstruction for recurrent patellofemoral instability provides reliable surgical outcomes. Patients with low-grade patella alta, trochlear dysplasia and those with slight elevated of TT-TG distance showed an increased rate of revision surgeries, re-dislocations and persistent joint instability compared to those in whom pathoanatomical risk factors are not present.

## Data Availability

This study does not contain any third material.

## Abbreviations

MPFL: Medial patellofemoral ligament
ROM: Range of motion
TT-TG: Tibial tuberosity-trochlea groove distance
SD: Standard deviation
OR: Odd ratio
N/R: Not reported
Semit: Semitendinosus
Ham: Hamstring
Synth: Synthetic
Add: Adductor
Quad: Quadriceps
